# Rare co-occurrence of multiple sclerosis and Wilson’s disease – case report

**DOI:** 10.1186/s12883-022-02691-5

**Published:** 2022-05-16

**Authors:** Katalin Despotov, Péter Klivényi, István Nagy, Attila Pálvölgyi, László Vécsei, Cecília Rajda

**Affiliations:** 1 Department of Neurology, University of Szeged, Faculty of Medicine, Szeged, Hungary; 2 Faculty of Medicine, Department of Internal Medicine I, University of Szeged, Szeged, Hungary; 3 MTA-SZTE Neuroscience Research Group, Szeged, Hungary; 4 Faculty of Medicine, Interdisciplinary Excellence Center, University of Szeged, Szeged, Hungary

**Keywords:** Multiple sclerosis, Wilson’s disease, Co-morbidity, Point mutation, Case report

## Abstract

**Background:**

Wilson’s disease is a hereditary disorder of copper metabolism resulting mainly in hepatic, neurological, and psychiatric symptoms. Multiple sclerosis (MS) is an immune-mediated demyelinating disease affecting the central nervous system (CNS). The co-occurrence of these two, although not unheard of in literature, is still considered to be very rare and can give rise to diagnostic difficulties. Also, comorbidity in MS highly influences quality of life and disease progression, which makes the timely diagnosis and treatment of these conditions essential.

**Case presentation:**

The aim of this study is to present a patient exhibiting symptoms of both MS and Wilson’s disease, as well as to conduct a detailed review of previously reported cases. The patient’s neurological symptoms (sensory disorder) as well as MRI and CSF findings were characteristic for MS. The diagnosis of MS preceded that of Wilson’s disease and was relatively mild in course. Currently, the patient receives cladribine as an immunomodulatory treatment after escalation from glatiramer acetate therapy. Apart from one episode of acute hepatic decompensation, during which transfusion, albumin supplementation and diuretic treatment was necessary, Wilson’s disease manifested as chronic impairment of liver function. The diagnosis of Wilson’s disease was established by the analysis of serum coeruloplasmin levels, histological examination and genetic findings. Continuous oral penicillamine therapy led to the slow normalization of hepatic function and significant amelioration of the patient’s symptoms. Correlating with cases previously reported, the course of MS was relatively mild, and like in three out of four other known cases, the symptoms of Wilson’s disease were mostly restricted to hepatic dysfunction.

**Conclusion:**

The case presented in our report is similar to those reported before. The co-occurrence of the two diseases seems to be more a coincidence than a sharing of common factors in their pathogenesis; however, they are considered to influence one another. Regarding rare co-occurrences such as this one, every new case is of high importance, as it enables a better evaluation and understanding of the clinical presentations that are more characteristic of these cases, thus aiding the estimation of disease course as well as possible therapeutic choices.

## Background

Wilson’s disease is a hereditary disorder of copper circulation and excretion, resulting in the accumulation of copper in the liver, brain, cornea, kidney, and other organs, mostly manifesting in hepatic, neurological, and psychiatric symptoms. The disease shows an autosomal recessive line of inheritance, and it is associated with mutations of the *ATP7B* (Adenosine-Triphosphatase-Copper Transporting Beta Polypeptide) gene [1, 2]. Multiple sclerosis (MS) is an immune-mediated demyelinating disease affecting the central nervous system, and it is characterized by symptoms disseminated in space and time [3]. Though the etiology of MS is not clearly understood, it is thought to be of multifactorial origin, and there are several suspected risk factors such as genetic susceptibility or viral infections [4–6].

Comorbidity in MS highly influences quality of life and disease progression, which makes the timely diagnosis and treatment of these conditions essential. Here we present a patient with the co-occurrence of MS and Wilson’s disease. Although not unheard of in literature [7–9], such constellation is still considered to be very rare and can give rise to diagnostic difficulties as well as affect therapeutic choices.

## Case presentation

A 33-year-old female administrator, who gave her written informed consent for publication, was admitted to our hospital in 2013. She was previously diagnosed with hyperprolactinaemia, due to which she received bromocriptine treatment. Investigations concerning family history revealed no relevant immunological or genetic conditions.

**Fig. 1:**
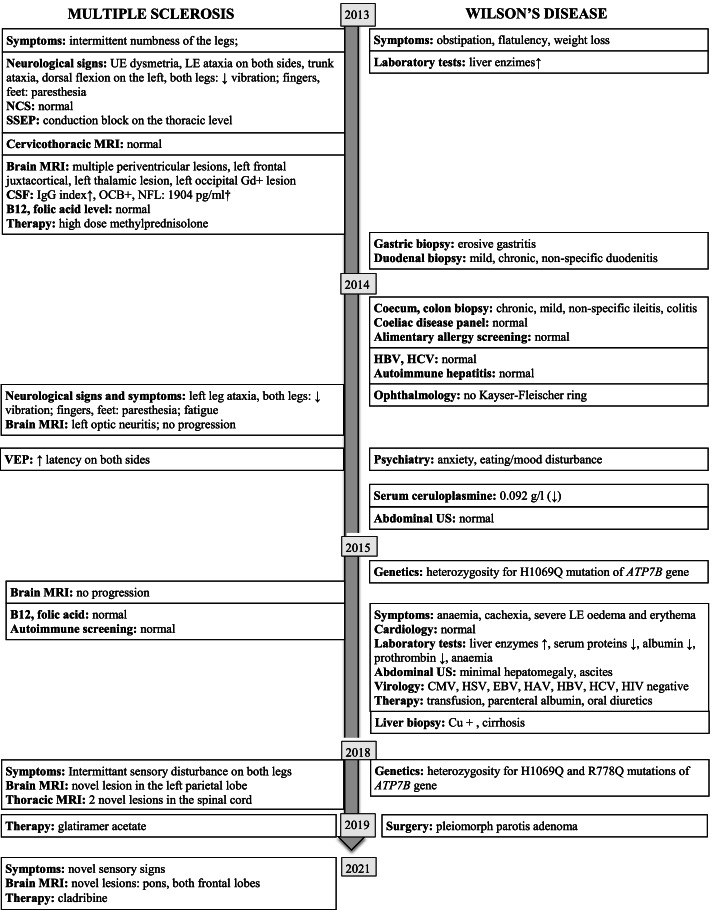
Disease course of multiple sclerosis and Wilson’s disease with relevant diagnostic findings. The signs, symptoms, test results, and therapy are indicated on the timeline. Left to the timeline arrow are the findings regarding multiple sclerosis, right to the timeline arrow are the results of the gastrointestinal tract examination. *†Analyzed retrospectively in 2019. Abbreviations: LE – lower extremity, UE: upper extremity, NCS – nerve conduction study, NFL – neurofilament light chain, OCB – oligoclonal band, SSEP – somatosensory evoked potentials, VEP – visual evoked potentials, MRI – magnetic resonance imaging, US – ultrasound, “↑” – increased/elevated, “↓” decreased*

She presented with limb ataxia and tactile sensory loss in both legs, accompanied by paresthesia of the hands and feet as well as anxiety and dysthymia (Fig. 1).

**Fig. 2:**
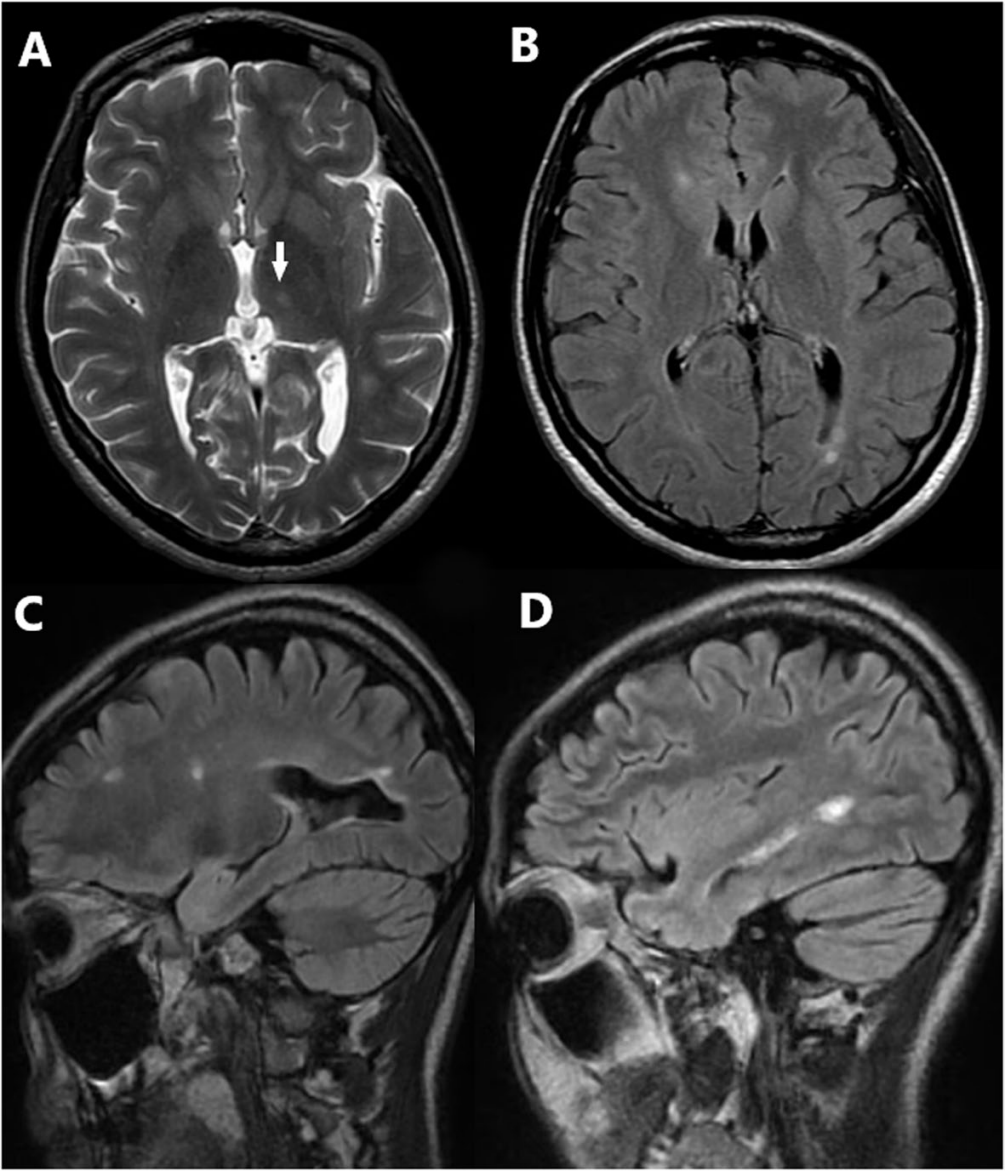
Brain MRI scans upon first admission. Axial T2 scan (**A**) shows a lesion in the left thalamus (full white arrow). Axial FLAIR (**B**) and sagittal FLAIR CUBE with gadolinium contrast media (**C-D**) scans reveal multiple periventricular white matter lesions typical for MS including a gadolinium enhancing lesion in the left occipital area, as well as a juxtacortical lesion in the left frontal region

**Fig. 3:**
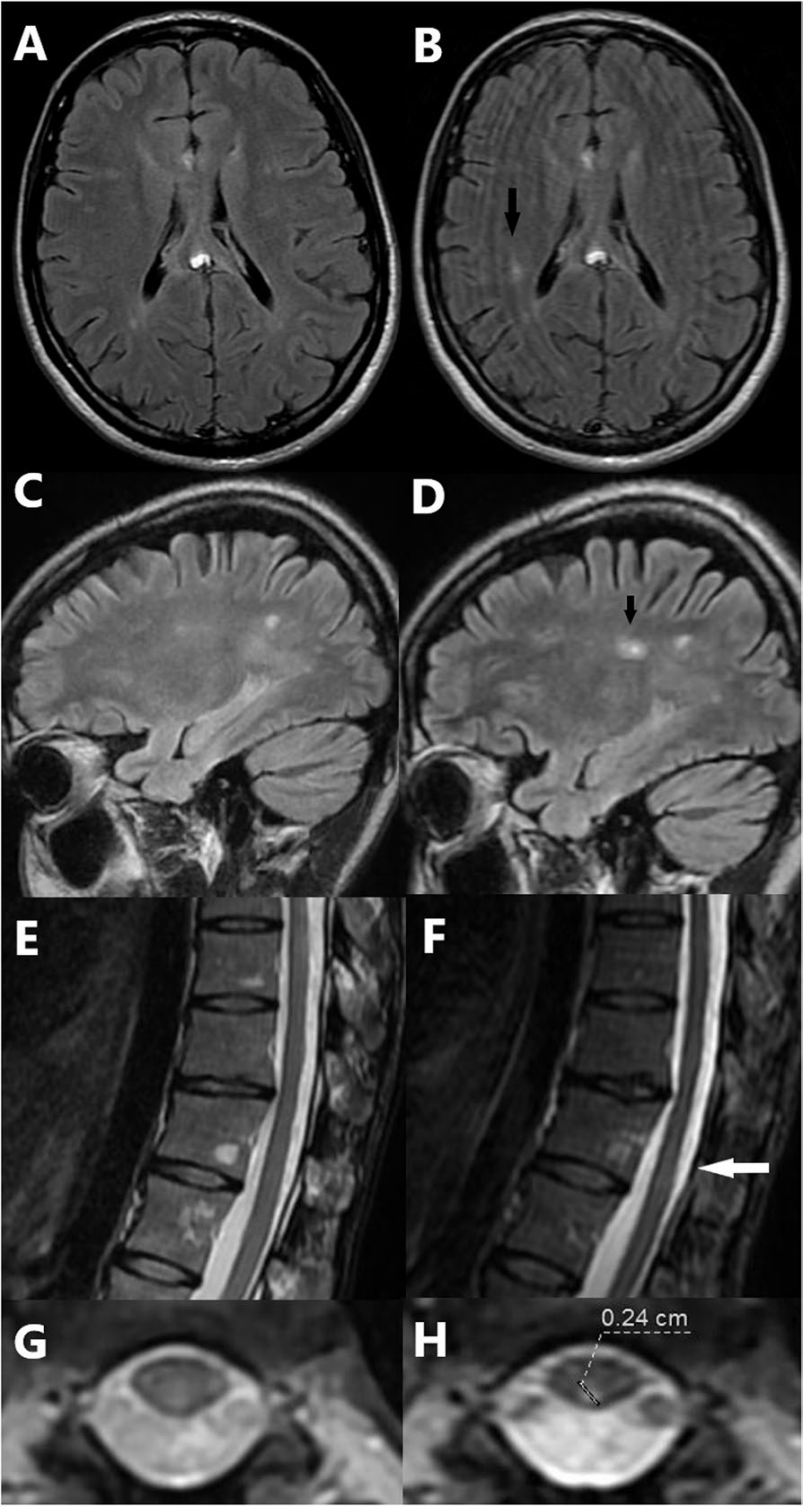
Brain and thoracic spine MRI scans: first admission (left) and second relapse (right). Axial FLAIR and sagittal FLAIR CUBE scans of the brain **(B**, **D)** as well as sagittal and axial T2 scans of the lower thoracic spinal region **(F**, **H)** taken in 2018 present novel lesions (black arrows, white arrow) compared to scans of the same quality and level from 2013 (**A**, **C**, **E**, **G**)

Nerve conduction study excluded peripheral neuropathy. Cervicothoracic spinal cord MRI showed no structural changes. Cranial MRI revealed bilateral, multiple periventricular white matter lesions, including a gadolinium enhancing lesion in the left occipital area, as well as a juxtacortical lesion in the left frontal lobe, and a lesion in the left thalamus (Fig. 2). In the cerebrospinal fluid (CSF), elevated IgG index (2.31; elevated when > 0.67) and oligoclonal bands (OCB) were detected. Cobalamine and folate levels were within the physiological range. The CSF neurofilament light chain level was 1904 pg/ml, retrospectively. Parenteral methyl-prednisolone therapy was initiated (3.5 g during the course of 4 days), leading to improvement in the neurological symptoms. Based on the clinical presentation and diagnostic findings, the criteria for dissemination in space and time were fulfilled in accordance with the McDonald Criteria of 2010 [10], and the diagnosis of multiple sclerosis was established. Despite international recommendations, at that time, the national therapeutic protocols did not support immunomodulatory treatment for patients with only one clinical event, while the patient herself was strongly opposed to long-term medication. For these reasons, at this point, disease modifying therapy could not be initiated. In 2014, a follow-up MRI scan indicated left optic neuritis with no progression of white matter lesions. Visual evoked potentials (VEP) revealed increased latency on both sides without clinical signs. In 2015, antibody levels indicative of systemic immunological diseases with potential CNS involvement were examined and found to be within normal range. Serologic tests ruled out a potential infectious etiology. After 5 years without clinical signs of disease activity, in 2018, a relapse occurred causing new, fluctuating sensory disturbances in both legs, with MRI scans showing multiple novel lesions in the spinal cord and one in the right parietal lobe (Fig. 3). At this time, EDSS was 1, the symbol digit modality test was 56, the 25 ft test was 5.75 and 5.40 seconds, and the 9 hole peg test was 23.2 and 19.1 seconds for the dominant right hand and 22.7 and 21.1 seconds for the left hand. After an elective surgery because of a pleiomorphic adenoma of the parotid gland, immunomodulatory treatment (glatiramer acetate) was initiated in 2019, which proved to be successful in preventing further relapses with no issues concerning adherence and tolerability.

Parallel to the neurological investigation, starting in 2013, a thorough gastrointestinal examination was conducted due to constipation and flatulency. Accompanying symptoms were anxiety, avoiding behavior, and eating problems. To evade uncomfortable situations at her workplace due to flatulency, the patient woke up at 4:00 in the morning to eat and fasted until the end of the working hours. She behaved the same way in the company of relatives. Consequently, she became underweight. Gastric, duodenal, coecum and colon biopsy showed chronic, mild and non-specific signs of inflammation. Coeliac disease and alimentary allergy screening was normal. Elevated liver enzymes were found in the serum, due to which several tests were performed to investigate an infective or immunological etiology, all yielding negative results. During the gastrointestinal follow-up in 2014, the suspicion of Wilsons’s disease was raised (Fig. 1). Serum ceruloplasmin levels were significantly decreased (0.092 g/l). Kayser-Fleischer ring was not detected. In 2015, she was hospitalized because of massive lower limb edema, hypoproteinemia, anemia, and ascites. Laboratory test results were consistent with hepatic decompensation. Abdominal ultrasound displayed hepatomegaly and ascites. Extended virological tests showed negative results. Liver biopsy proved chronic hepatic cirrhosis with copper accumulation. Genetic testing detected heterozygosity for the H1069Q and R778Q mutations of the *ATP7B* gene, both of which are missense point mutations classified as pathogenic. Based on these findings, the diagnosis of Wilson’s disease could be established [1]. There were no neurological symptoms typical of Wilson’s disease. At the time of severe hepatic decompensation, transfusion, parenteral albumin supplementation, and oral diuretic treatment were necessary. Subsequently, oral penicillamine therapy was initiated, which the patient continues until present day. This treatment led to the slow normalization of hepatic function and significant amelioration of the patient’s symptoms, including the disappearance of edema and ascites, as well as an improvement in her mood and appetite.

The patient was followed by a hepatologist monitoring liver function, performing laboratory tests and abdominal US as well as by a neurologist clinically. The patient displayed MRI progression (novel lesions in both frontal lobes as well as in the pons), and a relapse with loss of vibration in both lower limbs in 2021, which led to therapy escalation to cladribine. During the last examination, EDSS was 3 (due to sensory functional score), the symbol digit modality test result was 51, the 25 ft walking test was 6.13 and 5.57 seconds, and the 9 hole peg test was 20.24 and 19.4 seconds for the dominant right hand, while 24.96 and 23.28 seconds for the subdominant left hand.

## Discussion and conclusions

In this study, we present a patient diagnosed with both MS and Wilson’s disease. It is well recognized in literature that Wilson’s disease can cause a wide variety of CNS changes, the most frequent of which being the involvement of the basal ganglia, thalami and brainstem as well as the cerebral white matter, especially the corpus callosum [11–14]. While some of the white matter lesions seen in this case might be contributed to Wilson’s disease, gadolinium enhancement as detected in the left occipital area upon first admission, as well as spinal cord involvement occurring during the second relapse is not characteristic for Wilson’s disease [11, 14]. Considering that these lesions were detected in two different regions, approximately 5 years apart, in relation to two separate clinical events, the criteria of dissemination in space and time for MS could be fulfilled [3]. This diagnosis was also supported by the presence of OCBs in the CSF as well as the subclinical left side optic neuritis found during a follow-up MRI scan, neither of which is usually found in Wilson’s disease. In 2019 and in 2021, the decision for immunomodulatory treatment was made taking into consideration the potential hepatotoxicity of these therapies. During clinical and laboratory follow-up, both disease-modifying therapies proved to be a safe choice with no alteration of hepatic function, despite the just recently revealed potential hepatotoxicity of cladribine.

Apart from the one presented in this study, four other cases with coexisting Wilson’s disease and MS could be found in the literature (Table 1) [7–9]. All five known patients had CSF OCBs and MRI white matter lesions typical of MS with a mild disease course [7–9]. Pathological changes of the basal ganglia typical of Wilson’s disease were only detected in one case, reported by Yetkin et al. [9], while in two other cases there were thalamic lesions also often present in Wilson’s disease [7, 8, 11]. Four out of five patients were detected with heterozygosity for the H1069Q mutation of the *ATP7B* gene [7, 8]. This could be related to the fact that these four patients are from populations where this mutation is considered to have an especially high allele frequency: Poland (72%), Germany (47.9%) and Hungary (42.9%) [2, 7, 8, 15]. Previous studies indicate that the phenotype of Wilson’s disease shows considerable variability even amongst patients with the same genotype, and it has been suggested that the clinical presentation in compound heterozygotes carrying the H1069Q mutation is also determined by the type of mutation present on the second allele, as well as other genetic and environmental factors [16]. Only Patient I. reported by Dziezyc et al. exhibited neurological symptoms (hypomimia, sialorrhea, dysarthria, postural and intention tremor) which can be attributed to Wilson’s disease [7–9]. Apart from the patient with an onset at age 12 reported by Gunther et al., the other four patients were diagnosed with Wilson’s disease during adulthood preceded by the diagnosis of MS [7–9]. While the patient reported by Yetkin et al. had signs of encephalopathy of hepatic origin [9], it is questionable whether the personality changes reported by Dziezyc et al. or the mood disorders described in the present study can be regarded as a primary result of either of the diseases [7].

**Table 1:** Comparison of known cases with co-occurrence of MS and Wilson’s disease

	Patient I [7]	Patient II [7]	Patient III [8]	Patient IV [9]	Present Case
Age of onset	25 years	33 years	12 years	42 years	33 years
Extrapyramidal symptoms	hypomimia, sialorrhea, dysarthria, tremor, ataxia	–	–	–	–
Sensory symptoms	–	–	sensory disturbances in both legs	sensory disturbances on the right side of the face and body	sensory disturbances in both legs, four limb paresthesia
First diagnosed	MS	MS	Wilson’s disease	MS	MS
Course of MS	mild	mild	mild	mild	showing activity while on platform therapy
Psychiatric symptoms	behavior disorder	–	–	sings of encephalopathy	mood disorder, anxiety
MRI lesions (location)	periventricular WM, pons, cerebellum, thalamus	periventricular WM	periventricular WM, spinal cord	periventricular and subcortical WM, corpus callosum, spinal cord, basal ganglia	periventricular WM, thalamus, spinal cord
CSF	OCBs	IgG index ↑, OCBs	IgG index ↑, OCBs	OCBs	IgG index ↑, OCBs
Kayser-Fleischer ring	–	+	NA	+	–
Serum ceruloplasmin	↓	↓	↓	normal	↓
Genetic test (ATP7B gene)	1. allele: H1069Q 2. allele: Q1351X	1. allele: H1069Q 2. allele: A1135fs	1. allele: H1069Q 2. allele: c.2305insC, Codon 769, Exon 8	1. allele: R778L 2. allele: R778L	1. allele: H1069Q 2. allele: R778Q
Familial occurrence	1. sibling: MS 2. sibling: Wilson’s disease	1. sibling: Wilson’s disease	NA	none	none

*Abbreviations*: *MS* multiple sclerosis, *NA* not available, *OCB* oligoclonal band, *WM* white matter, “- “– not present, “+” – present, “↓” **–** decreased

While the prevalence of MS in Europe and North-America is around 100/100000 (ranging from 50 to 130/100000 in Europe), Wilson’s disease is considered to be a rather rare disease with a prevalence of 1:30.000 (3:100000) [2, 17]. In Hungary, the total number of Wilson’s disease patients is estimated to be around 300 [2]. Since the probability of a co-occurrence between the two diseases by coincidence is statistically low, it would be reasonable to consider some sort of connection. One possibility could be that rather than a co-existence, the cases described above might be atypical manifestations of one disease, though to our knowledge, so far, there is no evidence of a common pathogenic factor. Another possibility could be a connection at the genetic level. While the etiology of MS is not entirely understood, it is thought to be multifactorial, and there are several genetic factors that are suspected to play a role in susceptibility to the disease [5, 6]. Theoretically, it is possible that there is a linkage between one of these susceptibility genes and the *ATP7B* gene associated with Wilson’s disease. This theory could be supported by the fact that Patient I. reported by Dziezyc et al., had one sibling diagnosed with Wilson’s disease and another with MS [7] (Table 1). Despite the above-mentioned possibilities, the co-occurrence of the two diseases still seems to be more a coincidence than a sharing of common factors in their pathogenesis; however, the two conditions are considered to influence one another. Some authors [7, 9] suggest that the higher levels of copper in the central nervous system may further promote neurodegeneration in MS, while, on the other hand, elevated serum copper concentration, as well as D-penicillamine treatment has immunosuppressive effects, which could explain the relatively mild course of MS in these cases. The lack of means to examine the relationship and interactions between the two conditions could be considered a limitation of this case report.

Although the case presented in our report was similar to those reported before, due to the rareness of such co-occurrence, each new case is of high importance, as it enables us to better evaluate and understand which clinical presentations are more characteristic of these cases, providing aid in the estimation of disease course as well as in possible therapeutic choices, considering, for example, the potential liver toxicity of disease-modifying treatments in MS.

## Data Availability

The datasets generated during and/or analysed during the current study are available from the corresponding author on reasonable request.

## Abbreviations

*ATP7B* gene: Adenosine-Triphosphatase-Copper Transporting Beta Polypeptide
CNS: Central nervous system
EDSS: Expanded Disability Status Scale
MRI: Magnetic resonance imaging
MS: Multiple sclerosis
US: Ultrasound
VEP: Visual evoked potentials
WD: Wilson’s disease
